# Significance of postoperative membranous urethral length and position of vesicourethral anastomosis for short-term continence recovery following robot-assisted laparoscopic radical prostatectomy

**DOI:** 10.1186/s12894-022-01097-2

**Published:** 2022-09-07

**Authors:** Yasukazu Nakanishi, Shunya Matsumoto, Naoya Okubo, Kenji Tanabe, Madoka Kataoka, Shugo Yajima, Hitoshi Masuda

**Affiliations:** Department of Urology, National Cancer Center Hospital East, 6-5-1 Kashiwanoha, Kashiwa-shi, Chiba 277-8577 Japan

**Keywords:** Prostate cancer, Robot-assisted radical prostatectomy, Continence recovery, The position of vesico-urethral anastomosis, Membranous urethral length

## Abstract

**Background:**

We assess whether short-term recovery of urinary incontinence following robot-assisted laparoscopic radical prostatectomy (RARP) is associated with postoperative membranous urethral length (MUL) and position of vesico-urethral anastomosis (PVUA).

**Methods:**

Clinical variables including PVUA and pre-and postoperative MUL were evaluated in 251 patients who underwent RARP from August 2019 to February 2021. Continence recovery was defined as no pad or one security liner per day assessed by patient interview at least 6 months follow-up. Univariate and multivariate logistic regression analyses were used to assess variables associated with continence recovery at 3 months after the operation.

**Results:**

Continence recovery rates at 3 and 6 months were 75% and 84%, respectively. Lower BMI (< 25 kg/m^2^) (*p* = 0.040), longer preoperative MUL (≥ 9.5 mm) (*p* = 0.013), longer postoperative MUL (≥ 9 mm) (*p* < 0.001), higher PVUA (< 14.5 mm) (*p* = 0.019) and shorter operating time (< 170 min) (*p* = 0.013) were significantly associated with continence recovery at 3 months in univariate analysis. Multivariate analysis revealed that postoperative MUL (OR 3.75, 95% CI 1.90–7.40, *p* < 0.001) and higher PVUA (OR 2.02, 95% CI 1.07–3.82, *p* = 0.032) were independent factors for continence recovery. Patients were divided into 3 groups based on the multivariate analysis, with urinary continence recovery rates found to have increased in turn with rates of 43.7% versus 68.2% versus 85.0% (*p* < 0.001) at 3 months.

**Conclusions:**

PVUA and postoperative MUL were significant factors for short-term continence recovery. Preservation of urethral length might contribute to continence recovery after RARP.

## Background

Radical prostatectomy (RP) is the principal surgical treatment for localized prostate cancer. The aim of RP is to achieve oncologic and functional outcomes, including urinary continence preservation [1]. Reported percentages of patients with post-operative incontinence range from 6 to 20% [2–4]. Although the recent introduction of robot-assisted radical prostatectomy (RARP) has been reported to allow for more accurate anatomical information on peri-prostatic structures, postoperative continence outcomes have not improved compared to open radical prostatectomy (ORP). According to a randomized clinical trial, there was no difference in functional outcomes between RARP and ORP, with a slight difference in biochemical recurrence rates in favor of RARP [5]. A Swedish LAPPRO trial also reported the slight benefit of RARP over ORP regarding erectile dysfunction but not urinary continence or oncologic outcomes [6]. Even in the era of RARP, urinary incontinence remains a problem that needs to be improved.

Several factors including clinical characteristics: obesity [7, 8], membranous urethral length (MUL) [7, 9], and surgical techniques; neurovascular bundle (NVB) preservation [10, 11], reconstruction techniques [12], have been addressed for early recovery of continence. Of these factors, MUL is an important factor that directly correlates with the functional sphincter mechanism. Preservation of maximal MUL is important for postoperative urinary continence [13]. Particularly, postoperative MUL is considered to affect continence directly. In addition, bladder neck descent was recently reported to be associated with early continence outcome in retzius-sparing (RS) RARP [14]. Position of vesico-urethral anastomosis (PVUA) is also important, suggesting the importance of the surgical technique used. In particular, the relatively early recovery of urinary continence in RS-RARP is noteworthy and several studies have been reported [15, 16], and the assessment of continence recovery in the relatively early postoperative period is important.

In this study, we aimed to investigate the impact of postoperative MUL and PVUA on urinary continence recovery at a relatively early time point after RARP.

## Methods

### Study population

All procedures were approved by the institutional review board. A total of 251 patients who underwent RARP from August 2019 to February 2021 in National Cancer Center Hospital East (NCCHE) were analyzed retrospectively. All study protocols were approved by the Ethical Committee of NCCHE. All patients provided informed consent before surgery.

### Surgical technique

Surgery was conducted only in patients with localized and locally advanced prostate cancer without distant metastasis. Procedures were performed by 6 surgeons including 4 novices of < 20 RARP experiences, all of which were supervised by a senior surgeon (H. M.) using the da Vinci Xi robotic system (Intuitive Surgical, Sunnyvale, CA, USA), basically via transperitoneal anterior approach. The extraperitoneal approach was chosen only for patients with past open abdominal surgery. The decision to perform nerve preservation was made based on the wishes of the individual patient and the judgment of the attending physician after explaining that it would contribute to preservation of erectile function and urinary continence, assuming that the tumor was not in close proximity to the NVB on MRI. In all cases, puboprostatic ligament preservation was routinely performed. Dissection of the prostatic apex was performed with a sharp and direct division of the membranous urethra at the level of the urethroprostatic junction [17]. Rocco stitch was used for posterior reconstruction of the Denonvillier’s fascia [18, 19]. Vesico-urethral anastomosis was performed by using a 3/0 ‘‘barbed’’ running suture, starting at 5 o’clock on the urethra and then proceeding clockwise. The anterior reconstruction was performed by suturing visceral to parietal layers of the endopelvic fascia to recreate the pubovesical ligaments [12].

The outcome measured was continence recovery at 3 months after catheter removal, as previously repored [20, 21]. Urethral catheter was removed between postoperative day 5 to 7. Continence recovery was defined as no pad or 1 security liner per day by self-report, which was obtained during outpatient visit at 1, 3 and 6 months after operation.

### Data collected

**Fig. 1:**
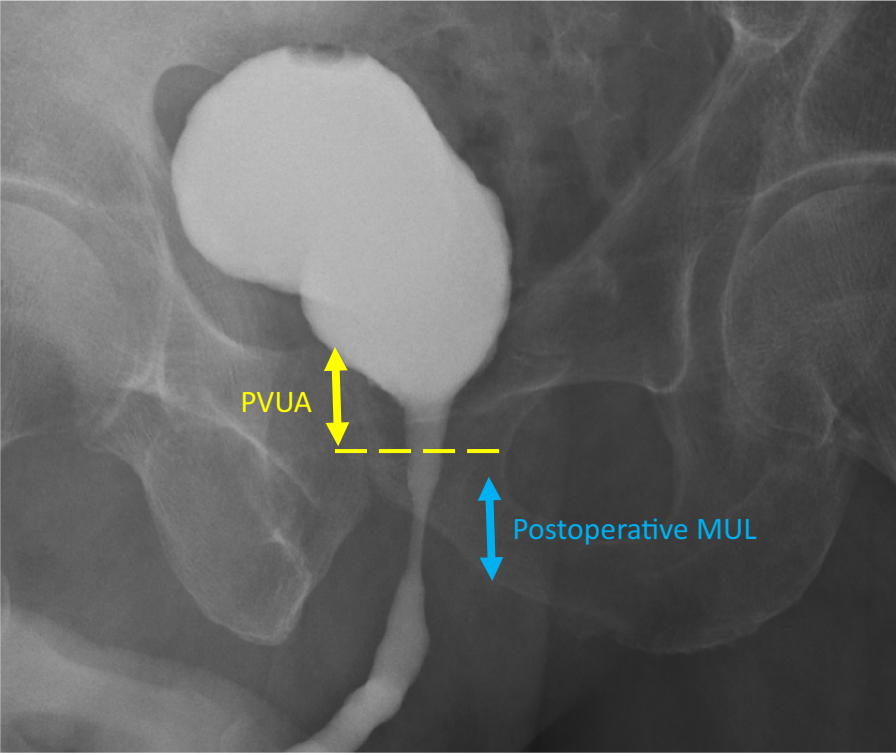
Cystourethrography showing the membranous urethral length (MUL, blue arrow) and position of vesico-urethral anastomosis (PVUA, yellow arrow)

The following clinical variables were evaluated: age, preoperative serum prostate-specific antigen (PSA) level, prostate volume, biopsy Gleason score (GS), clinical T stage, N stage, pre- and postoperative MUL, preoperative androgen deprivation therapy (ADT). Operation approach, neurovascular bundle (NVB) sparing, pelvic lymph node dissection (PLND), operation time and estimated blood loss (EBL) were recorded to assess perioperative parameters. The cut-off points of age, BMI, prostate volume, pre- and postoperative MUL, PVUA, operation time and EBL were set at 70-year-old, 25 kg/m^2^, 50 ml, 9.5 mm, 9 mm, 14.5 mm, 170 min and 200 ml by calculating the maximum Youden index with the highest value of “sensitivity − (1 − specificity)” in the receiver operating characteristics (ROC) analysis using continence recovery at 3 month after RARP as an endpoint, respectively. Risk classification was defined according to the European Association of Urology guidelines, in which clinical tumor stage (cT) 3–4 or clinical node stage (cN) 1 disease was classified as locally advanced disease [22]. ADT was offered as a part of multimodal therapy to most patients with locally advanced disease by the discretion of the outpatient physician. Preoperative MUL was estimated by measuring the length from the prostatic apex to the level of the urethra at the penile bulb in the midline sagittal plane of T2‑weighted magnetic resonance imaging of the prostate [9]. The postoperative MUL and PVUA were evaluated by the length of the sphincter portion which is measured the distance from the beginning of the urethral sphincter contraction to the release area in the absence of abdominal pressure, and vertical distance from the superior border of the pubic symphysis to the vesico-urethral anastomosis of the cystourethrography at the time of catheter removal (Fig. 1).

### Statistical analysis

Differences in the distribution of variables between groups were evaluated using a chi-square test or Fisher’s exact probability test. Uni- and multivariate logistic regression analyses were used to evaluate parameters associated with recovery of urinary continence after RARP at 3 months. A reduced multivariate model was developed using the stepwise backward method, in which the variable with the highest *p*-value was eliminated from each iteration of the multivariate analysis. Two-tailed *p* < 0.05 was regarded as significant. Statistical analyses were performed using JMP software version 13 (SAS Institute, Cary, NC, USA).

## Results

Characteristics of all 251 patients are summarized in Table 1. Median (range) age at the time of RARP and PSA were 72 (51–81) years and 8.2 (1.9–148) ng/mL. The numbers of patients with low- or intermediate-risk, high-risk, and locally advanced disease were 105 (41.8%), 83 (33.1%) and 63 (25.1%), respectively. Preoperative ADT was given to 97 (38.6%) patients. Pre- and postoperative MUL were 11.2 (5.8–18.8) and 10.1 (4.2–15.8) mm. Pathological T3 or ypT3 or more and positive surgical margin (PSM) were 55 patients (21.9%) and 59 patients (23.5%), respectively.

**Table 1:** Baseline demographics of the patients

Characteristics	Number (%) or median (range)
Age, year	72 (51–81)
BMI, kg/m^2^	23.2 (17.9–27.8)
PSA, ng ml	8.2 (1.9–148)
Prostate volume, ml	31.0 (7.9–109.8)
Biopsy Gleason score	
6	42 (16.7)
3 + 4	69 (27.5)
4 + 3	49 (19.5)
8	52 (20.7)
≥ 9	39 (15.6)
Clinical T stage	
T1c	11 (4.4)
T2a	133 (53.0)
T2b	2 (0.8)
T2c	42 (16.7)
T3a	53 (21.1)
T3b	10 (4.0)
Clinical N stage	
N0	243 (96.8)
N1	8 (3.2)
Risk classification	
Low or intermediate	105 (41.8)
High	83 (33.1)
Locally advanced	63 (25.1)
Preoperative ADT	
No	154 (61.4)
Yes	97 (38.6)
Preoperative MUL, mm	11.2 (5.8–18.8)
Postoperative MUL, mm	10.1 (4.2–15.8)
Approach	
Transperitoneal	230 (91.6)
Extraperitoneal	21 (8.4)
NVB sparing	
None	221 (88.0)
Unilateral or bilateral	30 (12.0)
PLND	
None	191 (76.1)
Standard or extended	60 (23.9)
PUVA	10.6 (− 5.3 to 25.2)
Operation time, min	168 (92–322)
EBL, ml	108 (0–1575)
Pathologic T stage	
ypT0	7 (2.8)
T2 or ypT2	189 (75.3)
T3 or ypT3	55 (21.9)
Surgical margin	
Negative	192 (76.5)
Positive	59 (23.5)

BMI, body mass index; PSA, prostate specific antigen; ADT, androgen deprivation therapy; MUL, membranous urethral length; NVB, neurovascular bundle; PLND, pelvic lymph node dissection; PVUA, position of vesico-urethral anastomosis; EBL, estimated blood loss

Continence recovery rates at 3-month and 6-month were 76.5% and 84.8%, respectively.

Lower BMI (< 25 kg/m^2^) (*p* = 0.040), longer preoperative MUL (≥ 9.5 mm) (*p* = 0.013), longer postoperative MUL (≥ 9 mm) (*p* < 0.001), higher PVUA (< 14.5 mm) (*p* = 0.019) and shorter operating time (< 170 min) (*p* = 0.013) were significantly associated with continence recovery at 3 months in univariate analysis. Multivariate analysis revealed that postoperative MUL (OR 3.75, 95% confidence interval [CI] 1.90–7.40, *p* < 0.001) and PVUA (OR 2.02, 95% CI 1.07–3.82, *p* = 0.032) were independent factors for continence recovery at 3 months (Table 2). In addition, only MUL was an independent factor for continence recovery at 6 months after surgery (OR 6.3, 95% CI 3.01–13.39, *p* < 0.001, not shown in the table).

**Table 2:** Univariate and multivariate analysis of continence recover after RARP at 3 month

Variables	Univariate analysis			Multivariate analysis			
	OR	95% CI	*p* value	OR	95% CI	Regression coefficient	*p *value
Age, year							
< 70	Ref						
≥ 70	0.64	0.34–1.22	0.17				
BMI, kg/m^2^							
< 25	Ref						
≥ 25	0.51	0.27–0.97	0.040				
Prostate volume*, ml							
≥ 50	Ref						
< 50	1.45	0.57–3.69	0.44				
Risk classification							
Low or intermediate	Ref						
High	0.65	0.35–1.18	0.16				
Locally advanced	0.62	0.33–1.19	0.15				
Preoperative ADT							
No	Ref						
Yes	0.74	0.42–1.35	0.33				
Preoperative MUL, mm							
< 9.5	Ref						
≥ 9.5	2.57	1.30–5.10	0.013				
Postoperative MUL, mm							
< 9	Ref			Ref			
≥ 9	3.79	1.94–7.42	< 0.001	3.75	1.90–7.40	0.66	< 0.001
Approach							
Transperitoneal	Ref						
Extraperitoneal	0.98	0.34–2.80	0.97				
NVB sparing							
None	Ref						
Unilateral or bilateral	1.26	0.48–3.25	0.63				
PLND							
None	Ref						
Standard or extended	0.63	0.33–1.22	0.17				
PVUA							
≥ 14.5	Ref			Ref			
< 14.5	2.05	1.11–3.80	0.019	2.02	1.07–3.82	0.35	0.032
Operation time, min							
< 170	Ref						
≥ 170	0.47	0.26–0.86	0.013				
EBL, ml							
< 200	Ref						
≥ 200	0.64	0.35–1.19	0.15				

BMI, body mass index; ADT, androgen deprivation therapy; MUL, membranous urethral length; NVB, neurovascular bundle; PLND, pelvic lymph node dissection; PVUA, position of vesico-urethral anastomosis; EBL, estimated blood loss; OR, odds ratio; CI, confidence interval

**Fig. 2:**
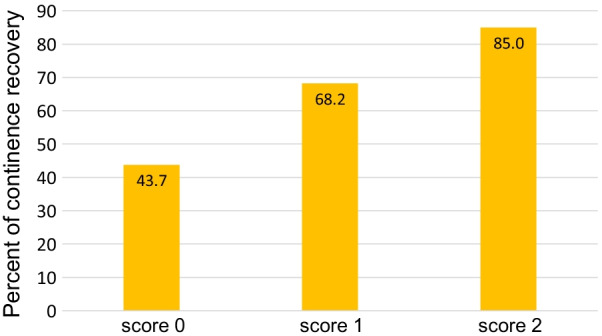
Percent of continence recovery at 3 months after robot-assisted radical prostatectomy by score

Based on the results of the multivariate analysis, we divided the patients into three groups according to the score predicting continence recovery at 3 months as follows: score = 1 (if PVUA < 14.5 mm) + 1 (if postoperative MUL > 9 mm). As shown in Fig. 2, the urinary continence recovery rates were increased in turn with rates of 43.7% versus 68.2% versus 85.0% (*p* < 0.001) at 3 months.

## Discussion

Recent advances in our knowledge of surgical anatomy have led to a better understanding of the mechanisms of urinary incontinence [23]. In terms of MUL, a systematic review and meta-analysis reported that preoperative MUL measurement is important for postoperative urinary continence [9]. In the present study, we demonstrated that PVUA and postoperative MUL measurement by cystourethrography are independently associated with continence recovery among men undergoing RARP, and the presence or absence of these factors significantly changes the relatively short-term urinary continence recovery rate.

Postoperative MUL as measured on cystourethrography has been reported to be the most important factor for recovery of urinary continence in the early postoperative period after RARP. It is greater with preservation of the neurovascular bundle, allowing for early recovery of urinary continence [24]. Postoperative MUL as measured on MRI has also been reported to be the most important predictive factor for recovery of urinary continence in the early postoperative period after RARP [21]. MRI measurements are accurate, but costly to perform in all patients postoperatively. Cystourethrography can be performed at the same time as vesico-urethral anastomosis evaluation in the early postoperative period and can predict the recovery of continence in almost all individual patients. Since a recent retrospective study, in which preoperative MUL was assessed by MRI and postoperative MUL was assessed by cystourethrography for the patients who underwent both ORP and RARP, reported that the superiority of postoperative MUL measurement over preoperative MUL for continence recovery [25], we consider postoperative MUL assessment to be an important test. Kojima et al. reported that the urethral sphincter system and periurethral support system are two main mechanisms of preserving urinary continence [26]. These sphincter components consist of an inner smooth muscle layer (longitudinal and circular smooth muscle) and a striated urogenital sphincter muscle (rhabdosphincter). In the current study, the MUL mainly evaluated the condition of the rhabdosphincter status. However, this sphincter is originally located from the prostate apex to the urethra at the level of the penile bulb [27], preservation of maximal urethral length is preferable during operation.

Our data also revealed that the importance of the PVUA for the recovery of urinary incontinence after RARP. This is because the height of the bladder neck position plays a significant role in incontinence. Olgin et al., first described the measurement method, bladder neck to pubic symphysis ratio, calculated by measuring the distance from the superior edge of the pubic symphysis to the bladder neck and dividing it by the total pubic symphysis height, in prediction of postoperative continence in patient received RARP [28]. Similarly for the postoperative outcome of RS-RARP, less bladder neck descent at postoperative cystography has been reported to be associated with better early continence outcomes compared to standard RARP [14]. The retzius-sparing technique preserves the anatomy of the pelvic floor and therefore the position of the vesicourethral anastomosis is higher than standard RARP. According to a systematic review and analysis, RS-RARP reported a statistically significant advantage in terms of continence recovery including relatively early period after operation [15]. A randomized study which first underwent long-term clinical trial reported that RS-RARP achieved a higher continence rate at 3 months postoperatively than the standard technique, which was muted at 12 months follow-up [16].

In our results, NVBP was not associated with urinary continence recovery, partly due to the small number of NVBP cases. The benefit of NVBP on urinary continence remains controversial. A large multicenter study of more than 3000 patients who underwent ORP or RARP indicated that the degree of bilateral NVBP predicted recovery of urinary continence 1 year after surgery, and that bilateral NVBP was beneficial for urinary continence [29]. On the other hand, a meta-analysis revealed that NVBP improves urinary continence in the first 6 months after surgery, thereafter, there is no difference in continence between men who had these nerves removed and those who had them saved [10]. In contrast, it has also been reported that it is not the nerve preservation itself, but the careful surgical technique that accompanies the nerve preserving operation that is key in improving recovery of incontinence [30]. Our results on nerve preservation also support this theory of careful dissection in some sense.

The current study demonstrated that when both higher PVUA and longer postoperative MUL are present, the recovery rate of urinary continence reaches 85% even with standard RARP. We showed that there is a clear difference in the recovery of urinary continence regardless of whether these items are aligned. Although it is not directly comparable, our method of PVUA evaluation is simpler than Olgin’s method. In addition, it is possible to evaluate the length of the urethra at the same time, so that incontinence can be predicted from two important factors. Intraoperative maximal MUL preservation contributes to the recovery of urinary incontinence in terms of both position and length.

At our institution, the number of cT1c cases were low and the rate of PSM was high. The reason for the former is that many of low-risk patients desired active surveillance as initial treatment. The latter reason was that many of patients underwent RARP were classified as intermediate to high risk, and even when high-risk patients requested NVB sparing, intra or inter-fascial NVB spearing was performed unless MRI-positive lesion contacted the prostatic capsule.

The present study has several limitations. Firstly, this study has the weaknesses characteristic of a retrospective design. Secondly, the number of patients was not so large. Thirdly, urinary continence status was evaluated based on the self-reported number of pads patients used, which is subjective. Instead, 24-h pad weight is desirable for accurate measurement [3]. Fourthly, the two factors obtained in the results of this study are postoperative factors. However, they are factors that can be determined at the time of removal of the urinary catheter, which is relatively early after surgery, so they are highly useful in actual clinical practice. Fifthly, since the PVUA is measured vertically from the suprapubic margin, it basically does not affect the angle of rotation during cystourethrography, but the possibility of a slight error cannot be denied. Sixthly, it is possible that the results of the analysis did not reflect nerve preservation due to the small number of cases. Finally, there were multiple surgeons with varying years of experience. The surgeon's learning curve in RARP has been suggested to affect the recovery of incontinence [31]. However, all surgeons used the same technique with little variability. In particular, when inexperienced surgeons perform surgery, all procedures are performed under the supervision of a senior physician in all cases to minimize differences in skill at our institution, and we believe that evaluating the results of multiple surgeons is realistic.

## Conclusions

PVUA and postoperative MUL were significant factors for short term continence recovery. Preservation of MUL for as long as possible might be important in determining favorable outcome of postoperative urinary continence.

## Data Availability

The datasets used and/or analyzed during the present study are available from the corresponding author on reasonable request.

## Abbreviations

ADT: Androgen deprivation therapy
CI: Confidence interval
EBL: Estimated blood loss
GS: Gleason score
MUL: Membranous urethral length
NVB: Neurovascular bundle
ORP: Open radical prostatectomy
OR: Odds ratio
PSA: Prostate-specific antigen
PLND: Pelvic lymph node dissection
PVUA: Position of vesico-urethral anastomosis
RARP: Robot-assisted laparoscopic radical prostatectomy
